# The origin of plasma neutrophil gelatinase-associated lipocalin in cardiac surgery

**DOI:** 10.1186/s12882-019-1380-4

**Published:** 2019-05-22

**Authors:** Arie Passov, Liisa Petäjä, Marjut Pihlajoki, Ulla-Stina Salminen, Raili Suojaranta, Antti Vento, Sture Andersson, Ville Pettilä, Alexey Schramko, Eero Pesonen

**Affiliations:** 1 Division of Anaesthesiology, Department of Anaesthesiology, Intensive Care and Pain Medicine, University of Helsinki and Helsinki University Hospital, Haartmaninkatu 4, PO BOX 340, FIN 00029 HUS Helsinki, Finland; 2 Children’s Hospital, Pediatric Research Center, University of Helsinki and Helsinki University Hospital, Stenbäckinkatu 9, PO BOX 347, FIN 00029 HUS Helsinki, Finland; 3 Department of Cardiac Surgery, Heart and Lung Center, University of Helsinki and Helsinki University Hospital, Haartmaninkatu 4, PO BOX 340, FIN 00029 HUS Helsinki, Finland; 4 Division of Intensive Care Medicine, Department of Anaesthesiology, Intensive Care and Pain Medicine, University of Helsinki and Helsinki University Hospital, Haartmaninkatu 4, PO BOX 340, FIN 00029 HUS Helsinki, Finland

**Keywords:** Neutrophil gelatinase-associated lipocalin (NGAL), Cardiac surgery-associated acute kidney injury, Acute kidney injury, Neutrophil activation, Biomarkers

## Abstract

**Background:**

Acute kidney injury (AKI) is common after heart surgery. Neutrophil gelatinase-associated lipocalin (NGAL) is produced in injured kidney. NGAL has been used as an early plasma biomarker for AKI in patients undergoing heart surgery. Neutrophils contain all isoforms (25-kDa, 45-kDa and 145-kDa) but the kidney produces almost exclusively the 25-kDa isoform of NGAL. We investigated first, whether there is association between NGAL and neutrophil activation, and second whether activated neutrophils are a significant source of circulating NGAL in plasma in patients undergoing cardiac surgery.

**Methods:**

Two separate patient cohorts were studied: 1) the “kinetic cohort” (*n* = 29) and 2) the “FINNAKI cohort” (*n* = 306). As NGAL is strictly co-localized with lactoferrin in neutrophils, NGAL and lactoferrin were measured with enzyme-linked immunosorbent assay in all patients. In sixty-one patients of the “FINNAKI cohort” Western blot was used to separate NGAL isoforms according to their molecular size. Mann-Whitney *U*, Kruskal-Wallis *H*, Pearson’s and Spearman’s tests were used as appropriate.

**Results:**

There was strong intraoperative association between NGAL and lactoferrin at all four time-points in the “kinetic cohort”. In the “FINNAKI cohort”, NGAL and lactoferrin concentrations correlated preoperatively (R = 0.59, *p* < 0.001) and at admission to the intensive care unit (R = 0.69, p < 0.001). At admission to intensive care unit, concentrations of NGAL and lactoferrin were higher in AKI than in non-AKI patients (NGAL: p < 0.001; lactoferrin: *p* < 0.029). In Western blot analyses, neutrophil specific 45-kDa isoform (median 41% [IQR 33.3–53.1]) and mostly neutrophil derived 145-kDa isoform (median 53.5% [IQR 44.0–64.9%]) together represented over 90% of total NGAL in plasma. Potentially kidney derived NGAL isoform (25-kDa) accounted for only 0.9% (IQR 0.3 – 3.0%) of total NGAL in plasma. There were no statistically significant differences in the distribution of NGAL isomers between AKI and non-AKI patients.

**Conclusions:**

Plasma NGAL during cardiac surgery is associated with neutrophil activation. Based on molecular size, the majority of circulating NGAL is derived from neutrophils. Neutrophil activation is a confounding factor when interpreting increased plasma NGAL in cardiac surgery.

## Background

Acute kidney injury (AKI) affects 25 to 30% of patients undergoing cardiac surgery and is associated with increased mortality [1, 2]. The diagnosis of AKI is based on the rise of creatinine and/or decrease in urine output over time [3]. For faster diagnosis of AKI, numerous biomarkers have been proposed*.* Neutrophil gelatinase associated lipocalin (NGAL) has been reported as an early urine and plasma biomarker of AKI in paediatric and adult cardiac surgery [4–7].

The origin of NGAL in urine and plasma is complex. NGAL exists in three molecular forms: (i) 25-kDa monomer; (ii) 45-kDa disulphide-linked homodimer; (iii) 145-kDa heterodimer consisting of a homodimer that is covalently attached to gelatinase [8, 9]. Only the monomeric and to lesser extent heterodimeric form is produced by the renal cells [10]. In contrast, neutrophils contain all isomers and the dimeric form is specific for neutrophils [8, 10, 11].

Clinical studies of plasma NGAL as a biomarker of AKI are based exclusively on immunologic methods [4, 6, 7, 12, 13]. However, antibodies against NGAL detect also other forms of NGAL besides the renal isoform [14]. Importantly, cardiopulmonary bypass (CPB) activates neutrophils that release their granule contents into the blood stream [15]. This poses a significant confounding factor in the interpretation of high NGAL values in plasma. It is possible to separate different NGAL isoforms according to their molecular size with Western blot analysis.

Our aim was to investigate if activated neutrophils are a significant source of plasma NGAL during and immediately after adult cardiac surgery. In neutrophils NGAL is mostly co-localized with lactoferrin (LF) [11]. Therefore, we first hypothesized that if plasma NGAL originates to a significant extent from polymorphonuclear neutrophils, there is association between plasma levels of NGAL and LF. Second, we hypothesized that in Western blot analyses, neutrophil derived isoforms would constitute a significant part of total NGAL in plasma.

## Methods

**Fig. 1:**
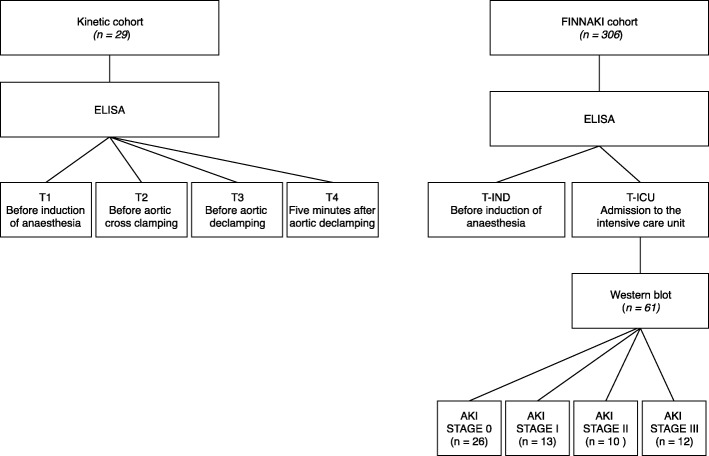
Title: Flow chart of the study cohorts. *ELISA – Enzyme-linked immunosorbent assay, AKI – Acute Kidney Injury*

Current publication includes data from two separate observational clinical studies (Fig. 1). The first study (“kinetic cohort”) comprised of twenty-nine patients undergoing aortic valve replacement surgery due to aortic valve stenosis. The exclusion criteria were as follows: other cardiac surgery in addition to aortic valve replacement during the same operation, coronary artery disease, left ventricular ejection fraction less than 30%, atrial fibrillation, systemic glucocorticoid medication or need for perioperative glucocorticoid substitution, immunosuppressive medication, insufficient cessation of anti-platelet (clopidogrel or ticagrelor less than 5 days), and anti-coagulation therapy (low molecular weight heparins less than 2 days).

The second study (“FINNAKI cohort”) consisted of heterogeneous cardiac surgical patients from the prospective observational study of cardiac surgery associated AKI (FINNAKI-HEART). Exclusion criteria of this study have been published previously [16]. From the 648 patients originally recruited to the FINNAKI-HEART study, postoperative plasma samples of 306 patients were still available for the present study. In both study cohorts, anaesthesia and CPB were conducted according to the institution’s standards, described previously in detail for both cohorts. [2, 17]. In brief, either propofol or etomidate together with an opioid (fentanyl, alfentanyl or sufentanyl) and rocuronium were used for anesthesia induction and sevofluran together with opioid infusion for anesthesia maintanence. Activated clotting time (ACT) target was > 480 s. Patients were treated with mild hypotermia of 33–34 °C except for the few patients undergoing aortic arch surgery with deeper hypothermia. Neither furosemide, ultrafiltration nor corticosteroids were used routinely.

In the kinetic cohort blood samples were drawn at four time points: (T1) before induction of anaesthesia; (T2) immediately before aortic cross clamping; (T3) immediately before aortic de-clamping; (T4) five minutes after aortic de-clamping. In the FINNAKI cohort blood samples were obtained before induction of anaesthesia (T-IND) and at admission to the intensive care unit (T-ICU) (Fig. 1). The latter time-point corresponds well to the previously used time-points for early prediction of AKI with plasma NGAL [4, 7, 12, 13]. Plasma was separated and stored in aliquots at − 80 °C.

The researchers that performed laboratory analyses were blinded to the patients AKI status. In both study cohorts, NGAL and LF were measured in all samples with commercial enzyme-linked immunosorbent assay (ELISA) kits (Hycult Biotech, Uden, The Netherlands).

For Western blot, a preliminary experiment was done using three random samples from the FINNAKI cohort. First, samples were mixed 1:4 with Laemmli sample buffer and protein content was quantified with commercial protein quantification assay (Marchery-Nagel GmbH, Düren, Germany). All samples were then investigated with non-reduced and reduced Western blot. For non-reduced Western blot, ten micrograms of protein was separated by electrophoresis using Mini-Protean TGX Stain-Free gels (Bio-Rad Laboratories, CA, USA) and transferred onto a polyvinylidene fluoride membrane (Thermo Fisher Scientific, MA, USA). Ten micrograms of commercial 25-kDa monomeric NGAL (Diagnostics Development, Uppsala, Sweden) was used as a positive control. Primary antibody used was a polyclonal rabbit anti-HNL-NGAL (Diagnostics Development, Uppsala, Sweden) at dilution of 1:1000. Normalization and quantification of the protein band intensity were carried out using Image Lab 6.0 software. For reduced Western blot the protocol was identical except that samples were supplemented with 1:100 β-mercaptoethanol and heated for 5 min at 95 °C before electrophoresis. The results of reduced Western blot demonstrated only the 25-kDa NGAL band. In contrast, in non-reduced Western-blot all three forms of NGAL were visible. Subsequently, the non-reduced Western blotting was used for research samples.

Western blotting was performed in 61 patients. Initially, all patients with AKI Stage 2 (*n* = 11) and AKI Stage 3 (*n* = 12) were selected for Western blot. One sample was missing and thus 10 patients with AKI Stage 2 and all patients with AKI Stage 3 were analyzed. We also analyzed 13 patients with AKI Stage 1 and 26 patients without AKI. The patients in both these groups (i.e. AKI Stage 1 and non-AKI groups) were first stratified according to the Acute Kidney Injury Risk Score [18] from highest to lowest. Patients were then selected with equal intervals from the highest to the lowest from each group by a researcher not involved in the laboratory analyses. We used this approach first in order to have a representative and unbiased aliquot of the AKI stage 1 and non-AKI patients. Second, since the AKI Injury Risk Score uses pre-operative data to predict AKI, having comparable patients across all AKI categories, the role of the perioperative renal injury is highlighted. Only samples retrieved at admission to ICU were used.

Pre- and post-operative plasma creatinine values were collected from the electronic laboratory database of the hospital. In order to represent a pure peri-operative AKI, the latest pre-operative creatinine was used as a baseline. Daily creatinine was registered during four post-operative days. AKI was defined according to the Kidney Disease: Improving Global Outcomes (KDIGO) guidelines based on the increase of creatinine (either an increase of ≥26.5 μmol/L during 48 h or 1.5 fold increase from baseline during four post-operative days) and initiation of renal replacement therapy (RRT) [3]. Daily creatinine at the third and the fourth post-operative day was also compared to the values of creatinine in preceding two days in order to account for the acute rise in creatinine as indicated by the KDIGO guideline [3]. The highest AKI stage was registered. Estimated glomerular filtration rate (eGFR) was calculated according to Modification of Diet in Renal Disease Study Group (MDRD) equation [19]. CKD was defined according to KDIGO guidelines based on eGFR [20]. For plasma creatinine peak delta change from baseline was calculated by subtracting the highest post-operative value during four postoperative days from the baseline value.

Pre-operative (the latest before surgery) and post-operative (first post-operative morning) white blood cell (WBC) counts were collected from the electronic laboratory database of the hospital.

Data are expressed as number (percent) or median and interquartile range (IQR). Data were analysed with SPSS Version 23 (IBM Corp, Armonk, New York, USA) and GraphPad Prism 7.00 (GraphPad Software, La Jolla, California, USA). Both studies were observational by nature. In the FINNAKI cohort, all appropriate patients with available blood samples were included. Therefore, power analysis was not conducted. Shapiro-Wilk test, Kolmogorov-Smirnov test and visual inspection of histograms were used for assessing data distributions. Logarithmic transformations were used for skewed data sets. If transformations failed to normalize the data distributions, non-parametric tests were used (Wilcoxon signed rank test for paired comparisons, Friedman test for testing differences as a function of time, Spearman’s rank test for bivariate correlations, Kruskal-Wallis *H* test and Mann-Whitney *U* test for differences between the groups). If logarithmic transformations yielded normal distribution profiles, parametric tests were used (Independent-Samples t-test, Paired-Samples t-test, Pearson’s test). The chi-square test was used for comparison of frequencies between the groups. Receiver operator characteristics (ROC) curves were generated for NGAL and LF and the area under the ROC curve (AU-ROC) was calculated for prediction of AKI. *P*-values less than 0.05 were considered statistically significant.

## Results

### Patient characteristics and procedure data

Patient characteristics and procedure data of study cohorts are presented in Table 1 (the kinetic cohort) and Table 2 (the FINNAKI cohort). In the kinetic cohort all patients underwent surgery with CPB. Chronic Kidney disease (CKD) was present in 7 (24.1%) of the patients.

**Table 1:** Kinetic cohort

Patient characteristics and procedure data	
Male gender	14 (48.3%)
Age (years)	66.0 (60.5–72.5)
Pre-operative creatinine (μmol/L)	76.0 (68.5–89.0)
Cardiopulmonary bypass time (min)	101 (85–114)
Aortic cross-clamping time (min)	70 (58–79)
Chronic Kidney Disease	7 (24.1%)
NGAL (ng/ml)	
*T1 - before induction of anaesthesia*	13.2 (11.3–16.7)
*T2 – immediately before aortic cross clamping*	9.3 (8.0–12.7)
*T3 – immediately before reperfusion*	25.2 (17.2–32.3)
*T4 – five minutes after reperfusion*	30.0 (17.8–41.3)
Lactoferrin (ng/ml)	
*T1 - before induction of anaesthesia*	62 (48–132)
*T2 – immediately before aortic cross clamping*	403 (311–541)
*T3 – immediately before reperfusion*	975 (598–1275)
*T4 – five minutes after reperfusion*	1177 (653–1377)

Data are median (interquartile range) or number (percentage). *NGAL* neutrophil gelatinase-associated lipocalin

**Table 2:** FINNAKI cohort. Patient characteristics and procedure data

	ALL [*N* = 306 (100%)]	NO-AKI [*N* = 238 (77.8%)]	AKI [*N* = 68 (22.2%)]
Age (years)	68 (60–75)	67 (58–74)	71 (64–78)**
Male gender	221 (72.2)	171 (77.4)	50 (73.5)
Co-Morbidity			
Insulin dependent diabetes mellitus	25 (8.2%)	16 (6.7%)	9 (13%)
Noninsulin dependent diabetes mellitus	55 (18.0%)	41 (17.2%)	14 (20%)
Hypertension	196 (64.1%)	146 (61.3%)	50 (73.5%)
Chronic Kidney Disease	40 (13.1%)	24 (10.0%)	16 (23.5%)**
Pre-operative LVEF less than 30%	11 (3.6%)	9 (3.8%)	2 (2.9%)
Surgery			
CABG only	152 (49.7%)	124 (52.1%)	28 (41.2%)
Valve-only	101 (33.0%)	79 (33.2)	22 (32.4%)
CABG and valve	38 (12.4)	24 (10.1%)	14 (20.6%)*
Other cardiac surgery	9 (2.9%)	8 (3.4%)	1 (1.5%)
Aortic rupture or dissection	6 (2.0)	3 (1.3%)	3 (4.4%)
Off-pump surgery	28 (9.2%)	26 (10.9%)	2 (2.9%)*
Cardiopulmonary bypass time^a^ (min)	103 (82–147)	102 (81–146)	104 (84–155)*
Aortic cross-clamping time^a^ (min)	72 (57–101)	74 (57–100)	70 (55–113)

Data are median (interquartile range) or number (percentage). ^a^only patients undergoing on-pump surgery used in calculations. *p < 0.05, **p < 0.01, ***p < 0.001 for AKI vs NO-AKI

*LVEF* left ventricular ejection fraction, *ICU* intensive care unit, *CABG* coronary artery bypass grafting, *AKI* acute kidney injury

In the FINNAKI cohort 28 (9.2%) of the patients were operated off-pump. In the FINNAKI cohort 40 (13.1%) patients had CKD before surgery (Table 2). In the FINNAKI cohort WBC counts were significantly higher in patients with AKI pre-operatively as well as at first postoperative day (Table 3).

**Table 3:** FINNAKI cohort. Laboratory analyses

	ALL [N = 306]	NO-AKI [N = 238 (77.8%)]	AKI [N = 68 (22.2%)]
Pre-operative creatinine (**μ**mol/L)	84 (72–97)	84 (72–94)	92 (75–109)**
Pre-operative NGAL (ng/ml)	21.5 (17.8–28.1)	21.0 (17.2–26.8)	22.6 (20.0–31.7)*
Pre-operative LF (ng/ml)	117 (72–189)	116 (72–192)	119 (70–167)
Pre-operative WBC count (10E9)	6.8 (5.7–8.2)	6.7 (5.6–8.1)	7.2 (6.2–9.1)*
NGAL at admission to ICU (ng/ml)	39.0 (28.6–52.6)	37.9 (27.3–48.7)	48.8 (35.4–61.7)***
LF at admission to ICU (ng/ml)	489 (338–651)	471 (336–622)	538 (362–856)*
WBC count at 1st postoperative day (10E9)	9.5 (7.9–12.1)	9.2 (7.8–11.8)	10.8 (8.2–13.0)*

Data are median (interquartile range) or number (percentage). WBC - white blood cell count

*p < 0.05, **p < 0.01, ***p < 0.001 for AKI vs NO-AKI. *NGAL* neutrophil gelatinase-associated lipocalin, *LF* lactoferrin, *WBC* white blood cell, *ICU *intensice care unit

### Acute kidney injury

In the kinetic cohort only one patient developed AKI (stage 1). None of the patients required RRT.

In the FINNAKI cohort 68 (22%) patients developed AKI. AKI Stage 1 was present in 45 (66.2%) of the cases. Severe, AKI Stage 2 and AKI Stage 3 occurred in 11 (16.2%) and 12 (17.6%) cases respectively. Of all AKI cases 16 patients (23.5%) had preoperative CKD. Of all AKI patients, 11 (16.2%) needed RRT during the first four post-operative days.

### Plasma NGAL and LF

**Fig. 2:**
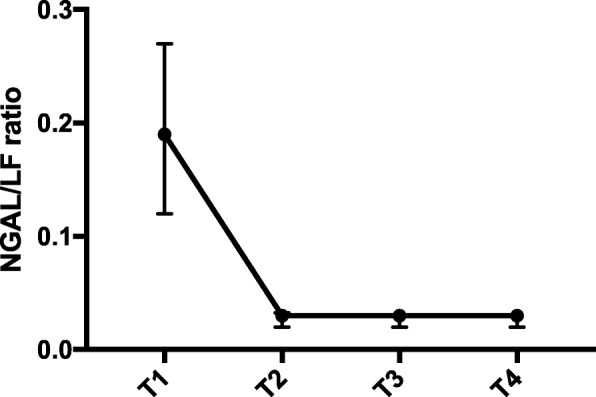
Kinetic cohort. NGAL indexed to LF (NGAL/LF ratio). *Data are median (dots) interquartile range (whiskers). NGAL – neutrophil gelatinase-associated lipocalin, LF – lactoferrin, T1 – before induction of anaesthesia, T2 – before aortic crossclamping, T3 – before aortic declamping, T4 – five minutes after reperfusion*

In the kinetic cohort, the median plasma concentrations of NGAL and LF (Table 1) changed significantly as a function of time [NGAL (*p* < 0.001); LF (p < 0.001)]. Despite this, NGAL indexed to LF (NGAL/LF-ratio) remained constant after the onset of CPB (Fig. 2). The concentrations of NGAL and LF correlated significantly at all time-points (T1: R = 0.51; *p* = 0.004, T2: R = 0.52; *p* = 0.012, T3: R = 0.72; *p* < 0.001, T4: R = 0.61; p < 0.001).

In the FINNAKI cohort, the median plasma concentrations of NGAL and LF (Table 3) were higher at admission to intensive care unit (ICU) compared to the pre-operative values [NGAL (*p* < 0.001); LF (p < 0.001)]. Also, the median concentrations of NGAL and LF were higher in AKI than in non-AKI patients at admission to ICU [NGAL (p < 0.001); LF (*p* < 0.029)] (Table 3). Patients undergoing off-pump surgery had significantly lower median NGAL and LF concentrations at admission to ICU [NGAL: 17.8 (14.3–23.2) ng/ml vs 40.9 (31.6–54.6) ng/ml, *p* < 0.001; LF: 223 (151–301) ng/ml vs 510 (372–685) ng/ml, *p* < 0.001].

**Fig. 3:**
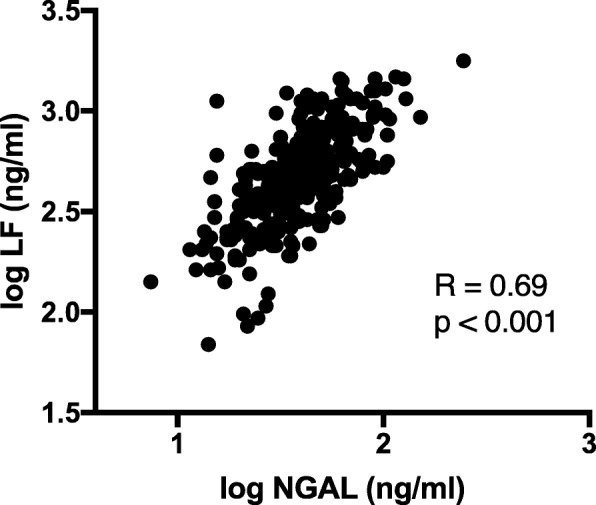
FINNAKI cohort. The correlation of NGAL and LF. *NGAL – neutrophil gelatinase-associated lipocalin, LF – lactoferrin*

Plasma NGAL and LF correlated significantly before surgery (R = 0.59, p < 0.001) and at admission to ICU (R = 0.69, p < 0.001, Fig. 3).

The AU-ROC for plasma NGAL and LF to predict AKI were 0.64 (95% CI 0.57–0.72; p < 0.001) and 0.58 (95% CI 0.51–0.66; *p* = 0.03) respectively.

Preoperative NGAL correlated statistically significantly with pre-operative eGFR in the kinetic cohort (R = − 0.40, p = 0.03) and in the FINNAKI cohort (R = − 0.22, p < 0.001). There was no correlation between eGFR and LF preoperatively (data not shown). In the FINNAKI cohort neither NGAL nor LF at admission to ICU had clinically meaningful correlation with peak-delta-change in plasma creatinine as a measure of severity of AKI (NGAL: R = 0.13, *p* = 0.02; LF: R = 0.14, *p* = 0.81).

In the FINNAKI cohort, both NGAL and LF at arrival to ICU correlated with aortic cross-clamping time (NGAL: R = 0.42, p < 0.001 and LF: R = 0.33; p < 0.001) and CPB time (NGAL: R 0.49, p < 0.001 and LF: R = 0.40, p < 0.001). Neither NGAL nor LF correlated with WBC counts.

### Western blot

**Fig. 4:**
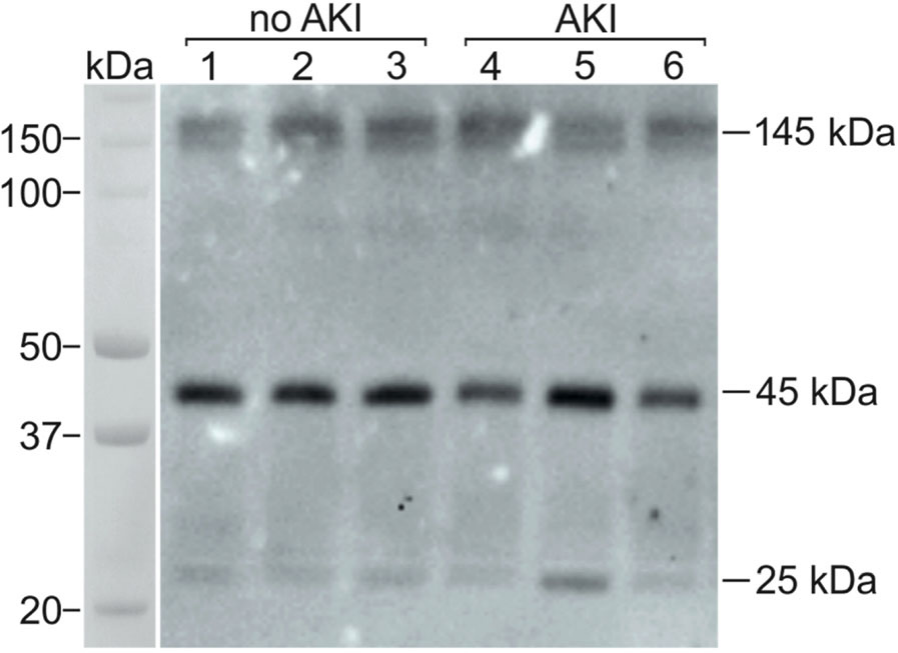
FINNAKI cohort. Representative Western blots of NGAL at admission to ICU. *NGAL – neutrophil gelatinase-associated lipocalin, AKI – Acute Kidney Injury*

**Fig. 5:**
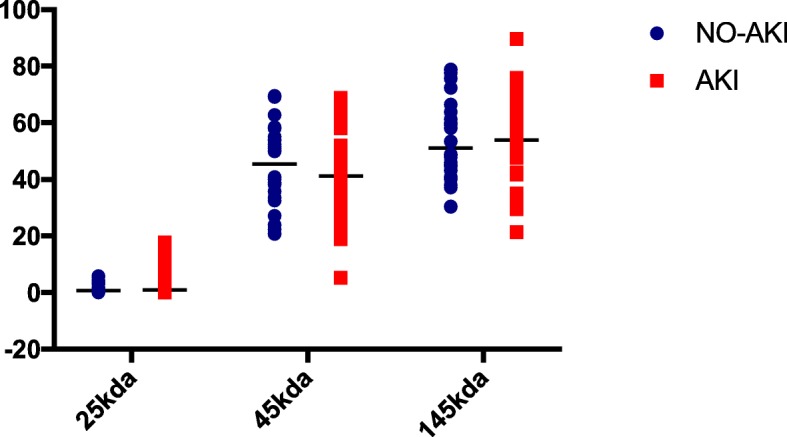
FINNAKI cohort. Distribution of NGAL isomers in patients with AKI and without AKI. *NGAL – neutrophil gelatinase-associated lipocalin, AKI – Acute Kidney Injury*

The non-reducing Western blots of 6 representative patients (3 with AKI and 3 without AKI) are shown in Fig. 4. The bands of neutrophil specific (45-kDa) and primarily neutrophil derived (145-kDa) isoforms were clearly visible in all blots. These two isoforms represented more than 90% of total NGAL in both AKI and non-AKI patients. Potentially kidney derived monomeric NGAL isoform (25-kDa) was visible in some of the patients. There was no statistical difference in the presence of 25-kDa isoform between patients with or without AKI (Fig. 5). Also, the AKI severity did not influence the presence of the renal isoform (Table 4).

**Table 4:** FINNAKI cohort. Quantification of NGAL isomers by Western blotting at admission to ICU

	ALL (*N* = 61)	NO AKI (*N* = 26)	AKI ALL (*N* = 35)	AKI STAGE-1 (*N* = 13)	AKI STAGE 2 (*N* = 10)	AKI STAGE 3 (*N* = 12)
Monomeric, 25-kDa (%)	0.9 (0.3–3.0)	0.7 (0.4–1.5)	0.9 (0.2–5.0)	1.5 (0.2–7.0)	0.7 (0.2–2.6)	1.0 (0.4–3.9)
Dimeric, 45-kDa (%)	41.2 (33.3–53.1)	45.4 (33.3–54.3)	41.2 (33.2–51.3)	41.1 (39.4–55.1)	39.4 (33.1–49.0)	41.2 (24.7–61.4)
Hetero-dimeric, 145-kDa (%)	53.5 (44.0–64.9)	51.2 (42.6–66.4)	53.8 (47.6–63.5)	52.4 (42.1–58.9)	56.6 (48.1–66.2)	58.2 (36.0–70.7)
NGAL at admission to ICU (ng/ml)	51.2 (37.2–77.3)	47.0 (35.3–79. 5)	53.4 (38.2–63.8)	57.0 (42.4–85.2)	47.3 (20.5–55.2)	54.0 (39.1–62.5)
AKI Risk-score	0.24 (0.16–0.41)	0.17 (0.10–0.30)	0.32** (0.18–0.47)	0.24 (0.20–0.43)	0.37 (0.19–0.49)	0.29 (0.18–0.52)

Data are median (interquartile range).™

*p < 0.05, **p < 0.01, ***p < 0.001 for AKI vs NO-AKI. *AKI* acute kidney injury

## Discussion

The primary finding of this study is, that during cardiac surgery with CPB, neutrophils are a major source of NGAL in plasma. In patients undergoing cardiac surgery, increased concentrations of NGAL in blood have been shown to be an early predictor of AKI [4, 6, 7]. NGAL is produced by the renal cells and it accumulates in large amounts in proximal tubular epithelial cells in experimental and clinical ischaemic kidney injury [21, 22]. Experimental studies demonstrate “back-leak” of NGAL from renal tubule into blood [23]. However, NGAL is also a constituent of polymorphonuclear neutrophils. Differentiation between neutrophilic and non-neutrophilic NGAL with immunological methods (ELISA, Triage® device, Architect® platform) that have been almost exclusively used in clinical NGAL research is difficult. Rather than detecting the renal isoform, different antibodies against NGAL bind to several isoforms of NGAL with different affinity [10]. Western blot analysis, however, separates different isoforms of NGAL according to their molecular size [24]. We chose a combined strategy based on first Western blot analysis and second co-localization of NGAL with LF in neutrophils.

Based on their time of formation, protein content and propensity for degranulation, neutrophil granules are dived into three major categories: secretory vesicles, primary granules also known as azurophilic granules and secondary granules [25]. During formation of the secondary granules, NGAL and LF are synthetizised concomittantly [26]. After synthesis NGAL and LF remain only in the secondary granules [11]. Majority of NGAL and LF is located in specific granules that are a subtype of secundary granules [27]. Upon degranulation of the specific granules, neutrophils release both these granule proteins to the surrounding medium and the released protein concentrations demonstrate high correlation with each other [9, 11].

Contrary to NGAL, expression of LF molecule is either low or virtually non-existent in the kidney [28–30]. Importantly, LF is not expressed in tubular epithelial cells [28]. Renal origin of circulating lactoferrin is thus unlikely. In contrast, LF is used as a marker of neutrophil activation and serves as an indicator of secondary granule degranulation [15, 31–33]. Furthermore, the concept of the present study, i.e. combined measurement of NGAL and LF has been applied previously to assess neutrophilic origin of NGAL [9, 11]. In our study, we investigated association of plasma concentrations of NGAL and LF in two different patient cohorts. Tight interrelationship between circulating NGAL and LF was observed. First, in the FINNAKI cohort, NGAL and LF correlated well pre- and post-operatively. Second, we used the kinetic cohort as a model of intraoperative neutrophil activation. In this smaller and more homogenic cohort we measured NGAL and LF preoperatively and at three intraoperative time-points. The pre-operative time-point T1 represents a state of minimal neutrophil activation while the time-points T2–4 during CPB represent a state of strong neutrophil activation. Initially LF increase was larger than increase in NGAL resulting in lower NGAL/LF ratio than preoperatively. However, subsequently, despite increase of NGAL of several folds during CPB (T2 to T4) in the kinetic cohort, NGAL/LF-ratio remained constant. This implies that the rise in plasma LF concentrations accounted for the proportional increase in plasma NGAL concentrations, i.e. there was no additional NGAL release apart from the one related to LF after the onset of CPB.

As our ELISA antibody recognized all isoforms of NGAL, correlation of plasma NGAL with neutrophil activation does not necessarily mean that NGAL would still originate from neutrophils. Therefore, we used Western blot which separates monomeric (25-kDa), homodimeric (45-kDa) and heterodimeric (145-kDa) forms of NGAL. However, two technical assumptions have to be met. First, an antibody that detects all three isoforms has to be used [34]. Second, preliminary experiments at our laboratory demonstrated that reducing of samples abolishes all but monomeric isoform. Thus, samples should be handled at non-reducing conditions to avoid destruction of di-sulphide bridges that bind monomers into homodimers and heterodimers. The non-reducing Western blot technique has been previously used in research of the origin NGAL in urine and tissue culture [10, 24]. Cultured human kidney epithelial cells have been shown to produce under both non-stressful and stressful conditions almost entirely the monomeric form of NGAL [10]. Only a very little fraction of heterodimeric form but no homodimeric form was produced. Neutrophils, on the other hand, produce all three isoforms of NGAL [8, 10, 11]. Our Western blot results show that the overwhelming majority of NGAL in plasma shortly after cardiac surgery with CPB is homo- and heterodimeric. The monomeric form was present only in small amounts. Statistically there was no difference between AKI and non-AKI patients in the presence of monomeric NGAL. Interestingly, among AKI patients there were some outliers with higher monomeric NGAL prevalence of up to 18%. One might speculate, that in patients with most severe AKI indeed there is some renal release of NGAL.

Literature offers indirect support for neutrophilic origin of plasma NGAL during cardiac surgery. First, CPB is a recognized pro-inflammatory condition with activation of neutrophils [15]. Longer duration of CPB is associated with higher circulating NGAL values [6, 35]. Consistent with this, the present study demonstrates correlations between NGAL, LF and cardiopulmonary bypass time and aortic cross-clamping time. Second, on-pump surgery has been associated with more profound neutrophil activation compared to off-pump surgery [36]. Likewise, in the FINNAKI cohort on-pump surgery was responsible for higher plasma NGAL and LF concentrations than off-pump surgery. This effect of on-pump surgery has also been demonstrated before [37]. Third, peri-operative methylprednisolone reduced plasma NGAL and LF similarly in pediatric cardiac surgery [38]. The latter is consistent with the fact that corticosteroids have been demonstrated to directly inhibit neutrophils [39].

The presence of significant plasma concentrations of neutrophilic NGAL in patients undergoing cardiac surgery raises a question, whether intrarenal activatation of neutrophils as a part of renal inflammation participate in the pathogenesis of AKI. Indeed, pathophysiological mechanisms of cardiac surgery associated AKI involve an inflammatory component [40, 41]. The role of activated neutrophils in the pathogenesis of AKI has been shown in experimental research [42]. However, only one study has addressed this issue specifically in clinical AKI associated with cardiac surgery [43]. In that paper, neutrophil activation in terms of CD11b expression was shown to be associated with post-operative AKI. In our study, lactoferrin concentrations were significantly higher at admission to ICU in patients that developed AKI indicating some association between AKI and neutrophil activation.

There are methodological strengths in our study. First, the ELISA antibody detected all NGAL isoforms. Therefore, the association of NGAL and LF cannot be attributed to measuring only the neutrophil derived NGAL isoform with ELISA. LF, on the other hand, has not been shown to be expressed in human tubular epithelial cells [28–30]. Second, for early prediction of AKI, previously used post-operative time window for NGAL measurement was used. Consequently, the AU-ROC for plasma NGAL in our study was similar to the one obtained in other large studies in adult cardiac surgery [13, 44]. Third, non-reducing Western blotting was performed and the antibody capable of detecting all isoforms of NGAL was used. Also, all patients with severe AKI were analyzed with Western blot. Furthermore, the AKI Stage 1 and non-AKI subgroups were stratified by Acute Kidney Injury Risk Score. This provided representative and comparable patient cohorts for all AKI categories. In other words, patients in all AKI categories had similar median risk for AKI. Therefore, the development of AKI was dependent on peri-operative factors. Thus, the possible differences between NGAL isomers between the groups, would have been due to perioperative kidney injury.

However, several potential limitaitions call for attention. First, these results cannot be directly generalised to other clinical conditions with elevated NGAL, i.e. sepsis and renal transplantation. Because CPB results in strong neutrophil activation, the present results cannot either be generalised to clinical circumstances with lower background neutrophil activation. In our patients, neutrophil activation, measured as plasma lactoferrin concentrations, was significantly lower in the off-pump than on-pump surgery. Despite this, the proportions of the 25-kDa NGAL isoform of the three off-pump patients with Western blot analyses were only 0.4% (non-AKI), 0.4% (AKI) and 5% (AKI). These proportions fall in the proportion range of the on-pump patients. The data of these few patients suggest that there is no major difference between off-pump and on-pump patients in this respect. Third, we did not analyse NGAL isoforms in urine. This was beyond the scope of the present study because it has already been studied in patients undergoing cardiac surgery [10, 14]. Indeed, monomeric NGAL (25 kDa) comprises significant part of NGAL in urine. However, in support of our findings, shortly after CPB, dimeric NGAL increases more than monomeric NGAL in urine. This might indicate either renal activation of neutrophils and/or renal excretion of dimeric NGAL shortly after CPB. Later this ratio is reversed, suggesting de novo synthesis of monomeric NGAL in the kidney (10). Finally, FINNAKI cohort included 40 patients with CKD. Neveretheless, exclusion of CKD patients did not change the results in any respect (data now shown).

## Conclusion

Plasma NGAL is an indirect and inaccurate marker of AKI shortly after CPB. During cardiac surgery with CPB plasma NGAL is mostly derived from activated neutrophils. Thus, neutrophil activation is a confounding factor when interpreting increased plasma NGAL in cardiac surgery with CPB. Activation of polymorphonuclear neutrophils seems to be modestly associated with AKI. The origin of NGAL in off-pump surgery and other clinical conditions like sepsis and renal transplantation remains to be investigated.

### Aknowledgements

Not applicable.

## Abbreviations

AKI: Acute Kidney Injury
AUROC: Area under the ROC curve
CPB: Cardiopulmonary bypass
ELISA: Enzyme-linked immunosorbent assay
ICU: Intensive care unit
IQR: Interquartile range
KDIGO: Kidney Disease: Improving Global Outcomes
LF: Lactoferrin
NGAL: Neutrophil gelatinase-associated lipocalin
NGAL/LF-ratio: Neutrophil gelatinase-associated lipocalin indexed to lactoferrin
ROC: Reciever operating characteristic
RRT: Renal replacement therapy
